# Depressive Symptoms in Late Pregnancy Disrupt Attentional Processing of Negative–Positive Emotion: An Eye-Movement Study

**DOI:** 10.3389/fpsyt.2019.00780

**Published:** 2019-10-31

**Authors:** Weina Tang, Ciqing Bao, Ling Xu, Jie Zhu, Wenqian Feng, Wenmiao Zhang, Cong Lin, Lan Chen, Qianqian Cheng, Penghao Ding, Meixi Zhou, Ying Bao, Xin Yu, Ke Zhao, Jincai He

**Affiliations:** ^1^School of Mental Health, Wenzhou Medical University, Wenzhou, China; ^2^Department of Obstetrics, First Affiliated Hospital of Wenzhou Medical University, Wenzhou, China; ^3^Department of Neurology, First Affiliated Hospital of Wenzhou Medical University, Wenzhou, China

**Keywords:** attention bias, depression, eye movement, emotional pictures, pregnancy

## Abstract

This study investigated biases for negative–positive information in component processes of visual attention (initial shift vs. maintenance of gaze) among women in late pregnancy with or without depressive symptoms. Eye movements were recorded while participants viewed a series of picture pairs depicting negative, positive, and neutral scenes. Initial orienting (latency and percentage of first fixation) and gaze duration were computed. Compared with neutral pictures, the group with major depressive symptoms (MDS) were less able to sense the positive emotion-related pictures and were over-responsive to negative emotion-related pictures. The group with suspicious depressive symptoms (SDS) had an attention bias toward both positive and negative emotion-related pictures. The group with no depressive symptoms (NDS) had an attention bias toward positive emotion-related pictures and had an initial attention avoidance tendency for negative emotion-related pictures. The initial gaze direction bias score for negative emotion-related pictures was positively correlated with the severity of depressive symptoms. Therefore, women with a risk of perinatal depression have a significant bias toward negative stimuli. Hypervigilant emotion processing during pregnancy may increase a woman’s susceptibility to depression during late pregnancy. Attention away from negative information or attention toward positive information may provide a way of buffering emotional responses.

## Introduction

Depression is common during pregnancy, with up to 70% of women reporting symptoms of depression during pregnancy and 10–16% fulfilling the criteria for major depressive disorder (1). Antenatal depression affects approximately 12% of women and is most prevalent in the second and third trimesters of pregnancy (7.4% in the first trimester; 12.8% in the second trimester; and 12.0% in the third trimester) (2). Additionally, some studies have indicated that depression is most common in the third trimester of pregnancy (3, 4). Accurate assessment and treatment are critical, due to the negative consequences of depression, including adverse familial, maternal, and fetal outcomes (5, 6), as well as maternal behaviors and thoughts related to suicide (7, 8). Furthermore, antenatal depression is the strongest predictor of postpartum depression (PPD) (9–11), and one-third of cases of PPD are reported to begin during pregnancy (12). It has been suggested that screening for depression during pregnancy is necessary, since early detection of depression during pregnancy can lead to the prevention of postnatal depression (9). Antenatal depression has been identified as a significant clinical issue but is a neglected component of care for women in late pregnancy (13, 14).

Although antenatal depression has a profound impact on maternal and fetal adverse outcomes, its risk factors and related mechanisms have not attracted the same attention as major depressive disorders, especially with respect to cognitive function. According to Beck’s cognitive theory (15), biases in the processing of emotional information play a crucial role in the occurrence and maintenance of depressive symptoms. An attention bias to negative information has been relatively consistently reported in patients with depression and appears to depend on the severity of the depression (16–18). Adults with major depression exhibit more errors in visual search tasks, show less accuracy in identifying positive emotions, and need a higher intensity of positive emotions to detect happiness (19–21). In addition, attentional bias of emotional stimulation has been recognized as a key mechanism of affective disorders, especially among patients with depression (18, 22, 23).

Data indicate that pregnant women have difficulty identifying emotional experiences and distinguishing between emotions and bodily sensations; this is considered a sign of alexithymia (19, 24, 25). Further, healthy mothers in the early postpartum stage exhibit changes in their capacity to identify visual sensory stimuli from their surrounding environment (26), which is thought to increase their vulnerability to depression (alexithymia predisposes people to depression (27, 28). In turn, depression can cause cognitive impairment (29). However, the field has yet to characterize the extent to which depression exerts systematic differences in affective information processing across the perinatal phases. Research on postnatal depression suggests that women with PPD and non-PPD are less able to recognize happiness and fear expressions, while compared with depressed non-partum women, those with PPD have poorer recognition of disgust and anger expressions (30). Additionally, women with PPD are reported to evaluate neutral baby faces as less neutral (31) and show higher amygdala activity when viewing negative emotional faces (32).

As for antenatal depression, studies show that depressive symptoms in early pregnancy disrupt attentional processing of infant emotion (33). For example, Pearson et al. used a disengagement paradigm and found that women who had depressive symptoms during pregnancy were faster at identifying the vertical line when the distracting image was a distressed (i.e., actively crying) infant face (33, 34). This was interpreted to reflect the fact that women with depressive symptoms during pregnancy have a diminished attentional bias toward distressed infant faces and are less able to perceive and respond appropriately to their infant’s emotional state (35). Notably, this may hinder the development of maternal sensitivity during pregnancy and disrupt mother–infant interactions independently of PPD (36).

Furthermore, evidence from a systematic review indicated that mothers with depression and anxiety are more likely to identify negative emotions (i.e., sadness) and are less accurate at identifying positive emotions (i.e., happiness) in infant faces. Additionally, women with depression may disengage faster from positive and negative infant emotional expressions (37). While these studies show that women with mood disorders during pregnancy have altered reactions to positive and negative stimuli, most of the research has relied on facial expressions, rather than social and nonsocial scenarios. Some of the studies have used tasks that consist of stimuli specific to motherhood experiences, particularly image stimuli of infant faces.

Thus, the goals of the present study were to extend previous research in two key ways (26, 33, 34, 37–40). First, given the high incidence of depression in late pregnancy, understanding the pre-depressive emotion risk markers in late pregnancy is crucial; in particular, identification of specific biases of attention would be highly beneficial for early prevention and intervention of PPD. This could also reduce the incidence of suicide and infant injury behavior. Second, we used eye tracking to directly detect attentional bias toward emotional stimuli (social or nonsocial scenes) in the context of free-viewing tasks, which allowed us to specifically examine potential biases in initial orienting of attention and sustained attention to emotional picture pairs depicting negative, positive, and neutral scenes.

Based on the theory and research reviewed above, we hypothesized that women with antenatal depressive symptoms would show biased attention toward negative emotional stimuli and would show an absence of positive bias in the maintenance of gaze indices when compared with women without antenatal depressive symptoms. Further, we hypothesized that the severity of depression would be related specifically to the magnitude of the maintenance of gaze indices in attentional biases to negative information. Given the lack of previous eye-movement studies of antenatal depression in this area, we did not make specific hypotheses with regard to initial and sustained attention to negative information.

## Methods

### Participants

The present study was part of a longitudinal research project on maternal antenatal and postpartum mental health conducted at the First Affiliated Hospital of Wenzhou Medical University between January 2018 and January 2019. The entire study was approved by the research ethics committee of Wenzhou Medical University, 2018-KY043.

The following inclusion criteria were applied: (a) right-handed; (b) between the ages of 18 and 45years; (c) pregnancy gestation of more than 28weeks; (d) normal color vision with naked eye or corrected vision; (e) signed the informed consent voluntarily. The exclusion criteria were as follows: (f) no serious pregnancy-related complications (preeclampsia, intrauterine growth restriction, or gestational diabetes); (g) no serious medical or neurological condition, and no substance dependence (except caffeine) in the past year; (h) no history of severe psychiatric conditions (e.g., psychotic or bipolar disorders) according to the fifth edition of the *Diagnostic and Statistical Manual of Mental Disorders* (DSM-5) (41).

### Measures

The sociodemographic information collected included age, education, marital status, income, and subjective occupational stress. The participants were also asked to report number of previous pregnancies and miscarriages, whether their current pregnancy was conceived naturally or by reproductive technology, whether their current pregnancy was planned, and how many biological children they wanted.

#### Assessment of Depressive Symptoms

##### EPDS

Depressive symptomatology during pregnancy was evaluated using the Edinburgh Postnatal Depression Scale (EPDS) (42, 43), a 10-item self-rating scale covering mood, fun, self-blame, anxiety, fear, insomnia, coping ability, sadness, crying, and self-injury. The EPDS is a self-report questionnaire that is commonly used (44). However, almost 70% of women exceeding the threshold of 10 satisfy the criteria for a diagnosis of depression (12). Research shows that the EPDS screening tool has been utilized most frequently in antenatal depression validation studies so far (3, 45–47) and is more easy to use in busy antenatal clinics because of its short scale items. The diagnostic thresholds most widely used for the EPDS are 9/10 and 12/13 (48, 49). The cutoff points of 9/10 are used as markers of possible minor depression (49), and scores >12 are associated with a diagnosis of major depressive disorder (42). The Chinese vision of EPDS has good validity. If being used to screen antenatal depression in women in the third trimester of pregnancy, the optimal critical value was 9.5, and the sensitivity and specificity were 0.786 and 0.834, respectively (50). In this study, assignment to each group was based on the EPDS scores. An EPDS score ≥13 was classified as major depressive symptoms (MDS), a score of 10 to 12 was classified as suspicious depressive symptoms (SDS), and an EPDS score ≤9 was classified as no depressive symptoms (NDS). In the current study, the internal consistency of the EPDS was very good (*α* = 0.91). All participants were invited to participate in clinical structural interviews. The standardized interviews were conducted by a trained interviewer.

### Eye-Tracking Paradigm

#### Materials

The stimuli consisted of 24 different paired pictures, each presented twice (48 trials in total). Each pair consisted of an emotional scene, negative or positive, paired with a neutral scene. When displayed on the screen, each picture measured 600×400mm with the centers of each picture 600mm apart. The pictures were selected from the International Affective Picture System (Center for the Study of Emotion and Attention, 1999) on the basis of their valence and arousal scores. The 12 negative pictures comprised images of people looking unhappy or crying. The 12 positive pictures comprised images of people enjoying activities and/or people with smiling faces. The 24 neutral pictures comprised images of people in emotionally neutral situations. The pictures were selected so that the three types differed significantly from each other in terms of valence score. The negative and positive picture sets did not differ significantly in arousal scores, although each differed from the neutral picture set in arousal. The positive, neutral, and negative pictures had mean valence scores (with SDs in parentheses) of 7.78 (0.34), 5.22 (0.30), and 2.30 (0.64) and mean arousal scores of 6.63 (0.34), 4.77 (0.81), and 6.84 (0.55), respectively.

#### Apparatus

Eye movements were monitored throughout encoding and retrieval using a Tobii TX300 with a sampling rate of 120Hz. All materials were presented on a 23-in. broadband display with a resolution of 1,024×768 pixels. The display was connected to a Lenovo microcomputer (model: Qi Tian 7A4000-N000). The technology used in this study was a noninvasive infrared-based binocular eye-tracking technique. Head movement was compensated for when calculating the direction and location of the gaze. The eye tracker was able to accommodate head movements within a 37cm×17cm plane at a 65cm viewing distance.

#### Procedure

During the free-viewing task, participants were seated on a sturdy chair in a comfortable position and viewed the screen from a distance of approximately 65cm. In order to ensure optimum gaze data quality, the eye tracker was calibrated for each individual (using a standardized 5-point calibration procedure) prior to the beginning of the attention task. Emotional and neutral pictures were presented equally as often on the left side as on the right side of the screen. The task consisted of eight practice trials, followed by a brief pause (3s) and then 48 experimental trials. Each trial of the attention task consisted of a fixation cross (presented centrally for 800ms), followed by a pair of scene pictures which were displayed for 1,500ms, and then a blank masking was displayed for 800ms. Participants were asked to look at the picture on the screen as if they were watching a television program (free viewing) but were instructed to return their focus to the central fixation cross whenever it appeared. The 48 stimulus pairs were presented in a new randomized order for each participant.

### Data Preparation and Dependent Variables

The emotional and neutral pictures presented on each trial were defined as areas of interest (AOI). Fixation data recorded with the eye tracker for each AOI were used to estimate sustained visual processing indices. Criteria for identifying an initial shift in gaze on each trial were as follows (51): (a) participant was fixated in the central region before picture onset; (b) eye movements occurred after at least 100ms and with a maximum fixation radius of 1° after picture onset and before picture offset (52); and (c) gaze was directed to either picture (left or right) rather than remaining at the central position during picture presentation. Eye-movement data were initially processed using Tobii Studio Software (2.0.6). Only participants with high-quality recordings (> 70%) were included in data analysis. Trials with missing data were removed from analyses. The groups did not differ in the number of trials removed: *Z*(48) = 0.78, *p* = 0.44.

Using gaze data collected by the eye-tracker system, four attentional indices (53) were extracted for the present study: (a) direction of initial gaze (i.e., percentage of first fixations on each AOI); (b) first-fixation latency (i.e., time elapsed until the first fixation occurs on each type of picture in each trial); (c) first-fixation duration (i.e., duration of the first fixation made on each type of picture in each trial); and (d) total fixation time (i.e., total time that each subject fixates on each type of picture in each trial).

#### Bias Scores

Researchers (54) assert that relative bias scores might be more relevant indicators of depression-associated processing of emotional information than absolute indices compared with emotional and neutral information. The relative bias scores of each attention index associated with each emotion category were calculated according to the guidelines.

For direction of initial gaze and total fixation time, we calculated the percentage of trials in which a subject fixed first on the emotional picture rather than on the neutral picture. A bias score greater than 50% indicates a preference to look at the emotional picture rather than the neutral picture, whereas a score lower than 50% indicates a bias in gaze toward the neutral picture. With regard to first-fixation latency and first-fixation duration, we calculated the bias scores by subtracting the corresponding value obtained for the neutral picture from the corresponding value obtained for the emotional picture, consistent with previous research (55). Bias scores of first-fixation duration greater than zero were interpreted as a bias toward emotional pictures, whereas those lower than zero indicated a preference for neutral ones. Bias scores of first-fixation latency were the opposite.

### Data Analysis

Differences in demographic and clinical characteristics were investigated using analysis of variance (ANOVA) and Kruskal–Wallis tests for continuous variables and chi-square tests of independence for categorical variables. Differences in initial orienting (direction bias score and latency bias score) and subsequent engagement (initial-fixation duration bias score and total gaze duration bias score) were analyzed using separate 3 (group)×3 (emotion picture) mixed factorial ANOVAs. The main effect between emotion pictures and groups was evaluated with the occupational stress as a covariate of no interest. One-sample *t* tests and least significant difference (LSD) follow-up tests were used for further analysis. A series of zero-order correlation analyses were conducted to explore the relationships between attentional bias indices and depressive symptoms. All tests were two-tailed with an alpha level of 0.05, unless otherwise stated.

## Results

### Group Characteristics

**Figure 1:**
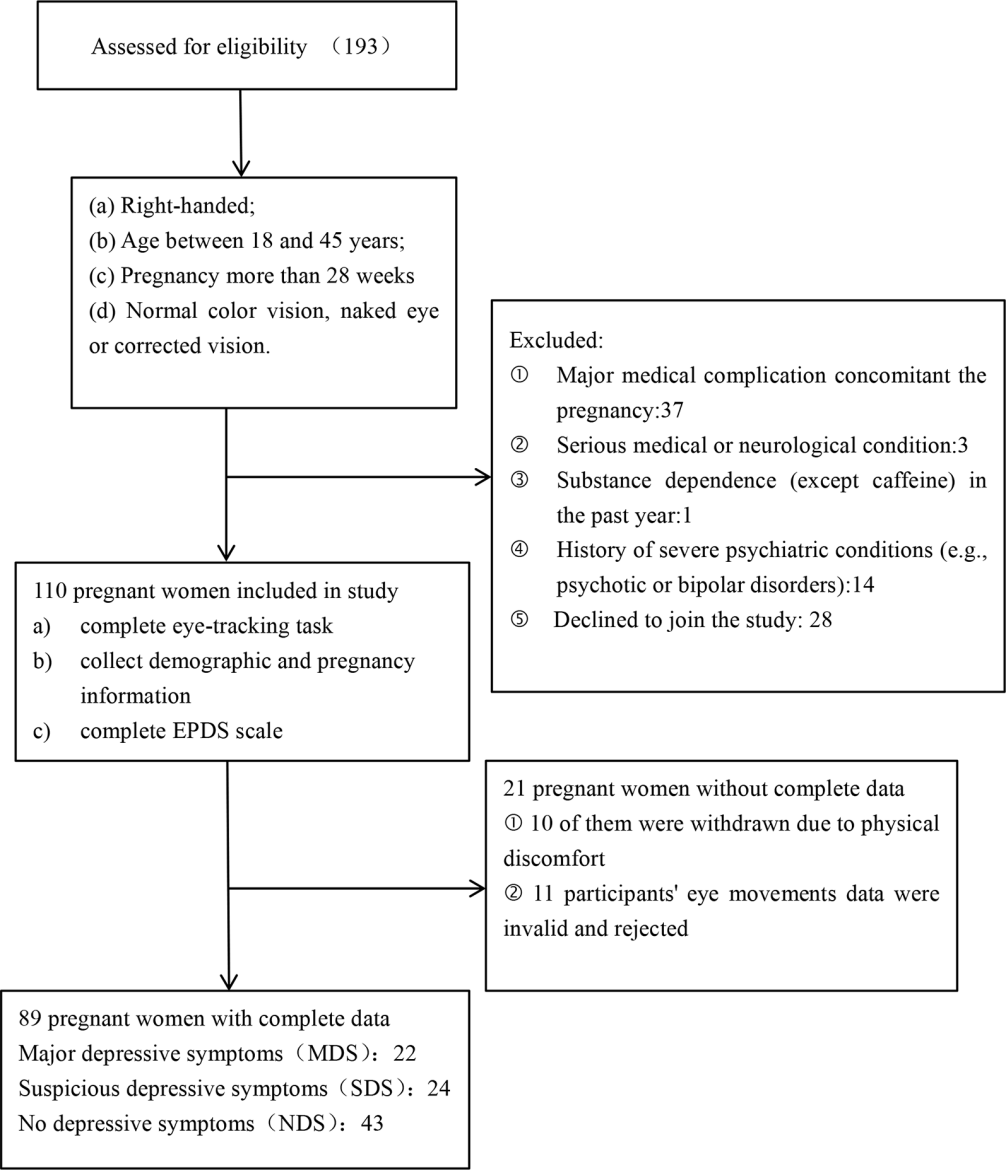
Study recruitment profile.

A total of 193 pregnant women were recruited for this study; of these, 110 pregnant women (gestation week: M = 36.03, SD = 1.45) who met the entry criteria were registered for participation (**Figure 1**). A tertile split was conducted on the participants’ scores on the EPDS scales. There were 22 participants in the MDS group (EPDS: M = 14.68, SD = 1.99), 43 participants in the NDS group (EPDS: M = 6.21, SD = 1.74), and 24 participants in the SDS group (EPDS: M = 10.50, SD = 1.10). In the end, 89 participants were included in the analysis of the stimuli processing data (see **Figure 1**). We used G*Power to estimate the sample size, which is a free power analysis program. The minimum total sample size calculated is 80, and the minimum sample size for each group calculated is 22, so the sample size of our study is within a reasonable range (56–60). Further details of the three groups can be found in **Table 1**.

**Table 1:** Differences in demographic and clinical characteristics among groups.

Characteristic	MDS (*N*=22)	SDS (*N*=24)	NDS (*N*=43)	*x*^2^/*Z*/*F*	*p*
Age	29.8±4.50	28.5±3.60	28.3±3.60	1.137	0.325
Occupational stress (%)				11.032	0.026*
Light	72.7	58.3	81.4		
High	27.3	41.7	18.6		
Exercise habits(%)				5.040	0.080
Yes	4.5	12.5	25.6		
No	95.5	87.5	74.4		
Childbirth history (%)				0.825	0.662
Primiparity	48.1	54.2	46.5		
Multiparity	59.1	45.8	53.5		
Educational level, median (IQR)	14 (8–15)	14 (8–18)	14 (8–18)	3.172	0.205
Gestational week, median (IQR)	36.3 (32.6–38.4)	36.6 (33.2–40.3)	36.4 (33.2–39.4)	1.103	0.576
EPDS, median (IQR)	14.68 (13–21)	10.5 (9–12)	6.21 (1–8)	75.695	0.000*

M, Mean; SD, standard deviation; MDS, major depression symptoms; SDS, suspicious depressive symptoms; NDS, non-depressive symptoms; IQR, interquartile range; EPDS, Edinburgh Postnatal Depression Scale.

^a^ Significantly more common than Non-Depressive Symptoms group, p < .001,.One-Way Anova:Post Hoc Multiple Comparisons.

^b^ Significantly more common than Suspicious Depressive Symptoms group, p < .001.

^c^ Significantly more common than Non-Depressive Symptoms group, p < .001.

* Significant difference between depressive symptom subgroups (p < 0.05).

### Attentional Processing of Emotional Information

#### Direction of Initial Gaze (Bias Score)

**Figure 2:**
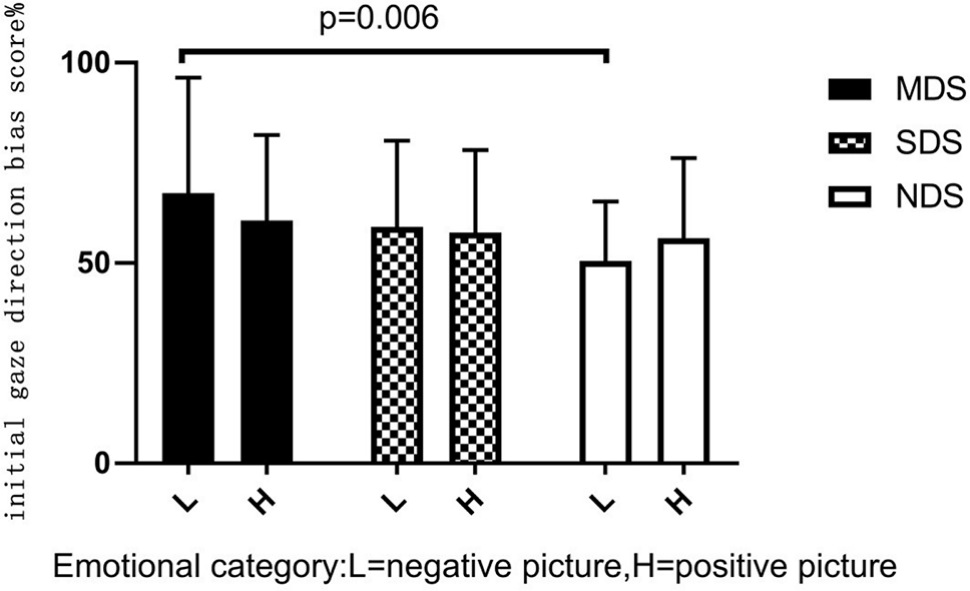
Initial gaze direction bias score (%) for emotional stimuli.

A 2×3 mixed-model ANOVA showed a nonsignificant group emotion interaction, *F*(2,86) = 1.428, *p* = 0.243, *η* ^2^ = 0.016, and a nonsignificant main effect for emotional category, *F*(1,86) = 0.072, *p* = 0.789, *η* ^2^ = 0.000. There was a significant main effect of group, F(2,86) = 3.437, *p* = 0.034, *η* ^2^ = 0.039. *Post hoc* LSD-T analyses indicated that the MDS group (*p* = 0.006) had a significantly higher initial gaze direction bias score for negative images than the NDS group (**Figure 2**). To explore this attentional pattern in more detail, we compared each bias score with a value of 50% (no bias). Analyses revealed a general bias for participants to look initially at positive or negative scenes rather than neutral scenes (see **Table 2**). These results indicate that initially, the MDS and SDS groups tended to fixate more frequently on the emotional scenes than on the neutral ones. Participants in the NDS group were more likely to direct their initial gaze to the positive picture.

**Table 2:** Statistics for the bias scores of the three group participants on the positive and negative pictures compared with the neutral pictures.

Bias scores	Group	Positive-neutral				Negative-neutral			
		M	SD	*t*/*Z*	*p*	M	SD	*t*/*Z*	*p*
Direction of initial gaze (%)	MDS	60.61%	21.39	3.289	0.002*	67.42%	28.86	4.005	0.000**
	SDS	57.63%	20.55	2.576	0.013*	59.02%	21.55	2.902	0.006*
	NDS	56.20%	20.01	2.874	0.005*	50.57%	14.83	0.359	0.721
First fixation latency (ms)	MDS	−113.58	162.28	−4.643	0.000**	−96.86	195.95	−3.279	0.002*
	SDS	−125.37	207.67	−4.183	0.000**	−57.56	182.24	−2.188	0.034*
	NDS	−98.59	179.74	−5.087	0.000**	−44.03	177.82	−2.296	0.024*
First fixation duration (ms)	MDS	58.52	176.43	2.200	0.033*	101.35	241.05	2.789	0.008*
	SDS	148.03	292.45	3.507	0.001*	167.53	307.58	3.774	0.000**
	NDS	98.19	180.60	5.042	0.000**	−0.41	245.355	−0.015	0.988
Total fixation time (%)	MDS	52.51%	8.96	1.856	0.070	54.99%	11.98	2.766	0.008*
	SDS	57.85%	13.06	4.162	0.000**	58.22%	13.27	4.293	0.000**
	NDS	55.64%	11.66	4.485	0.000**	51.18%	13.00	0.842	0.402

M, mean; SD, standard deviation; MDS, major depressive symptoms; SDS, suspicious depressive symptoms; NDS, nondepressive symptoms; t, statistical value of one-sample t-test; Z, statistical value of nonparametric test; *p < 0.05, **p < 0.01.

#### First-Fixation Latency (Bias Score)

A 2×3 mixed-model ANOVA showed a nonsignificant interaction between emotional category and group, *F*(2,86) = 0.411, *p* = 0.663, *η* ^2^ = 0.005; a non-significant main effect for group, *F*(2,86) = 2.115, *p* = 0.124, *η* ^2^ = 0.024; and a significant main effect for emotional category, *F*(1,86) = 6.568, *p* = 0.011, *η* ^2^ = 0.037. Further comparisons revealed that participants fixated faster on negative pictures than on positive pictures (*p* = 0.014). Comparisons with a no-bias criterion (zero) indicated that participants fixated faster on emotional rather than neutral pictures (see **Table 2**). Thus, these findings indicate not only that the three groups of participants directed their first fixation to the emotional picture earlier but also that the speed of detection of emotional pictures was significantly faster than that for neutral pictures.

#### First-Fixation Duration (Bias Score)

**Figure 3:**
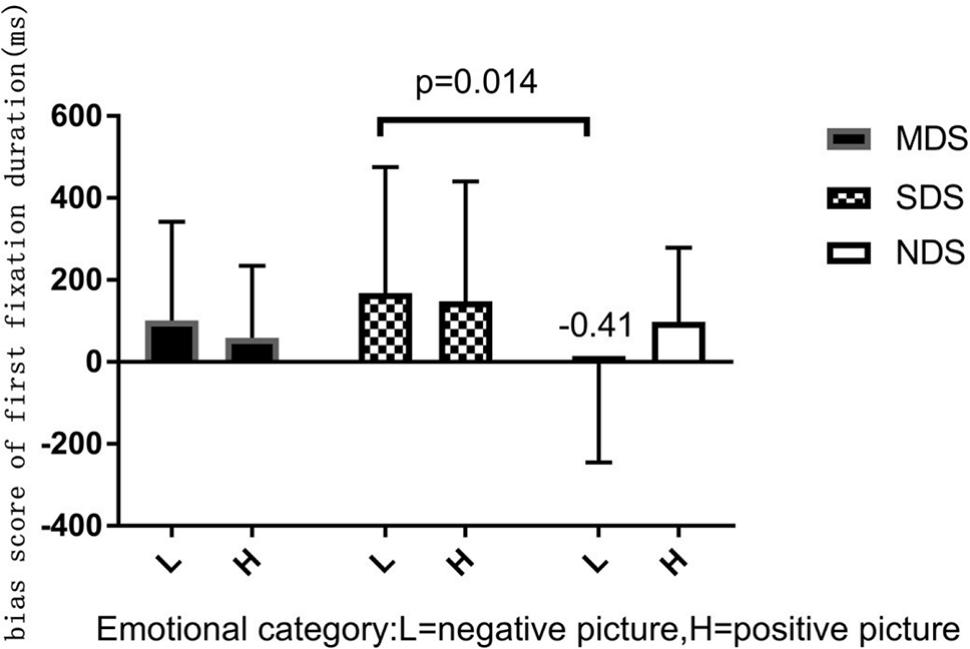
Bias score of first fixation duration (ms) for emotional stimuli.

A 2×3 mixed-model ANOVA showed no significant group emotion interaction, *F*(2,86) = 1.628, *p* = 0.199, *η* ^2^ = 0.019, and no significant main effect for emotional category, *F*(1,86) = 0.103, *p* = 0.749, *η* ^2^ = 0.001. There was a significant main effect of group, *F*(2,86) = 3.169, *p* = 0.045, *η* ^2^ = 0.036. *Post hoc* LSD-T analyses indicated that, compared with the NDS group, participants in the SDS group (*p* = 0.014) had significantly longer first-fixation durations on negative pictures (**Figure 3**). Comparisons with a no-bias criterion (zero) indicated that participants with depressive symptoms had a longer first-fixation duration on emotional rather than neutral pictures. However, the NDS group maintained a significantly longer fixation duration on positive images compared with neutral pictures. In addition, the NDS group had an initial attention avoidance tendency to negative images (bias scores = −0.41<0), although the difference was not statistically significant, *t*(42) = −0.015, *p* = 0.988 (see **Table 2**).

#### Total Fixation Time (Bias Score)

A 2×3 mixed-model ANOVA showed no significant group emotion interaction, *F*(2,86) = 1.411, *p* = 0.247, *η* ^2^ = 0.016, and no significant main effect for emotional category, *F*(1,86) = 0.080, *p* = 0.778, *η* ^2^<0.001. Further, there was no significant main effect of group, *F*(2,86) = 1.396, *p* = 0.250, *η* ^2^ = 0.016. Comparison of each bias score with a value of 50% (no bias) indicated that the bias scores of the three groups of participants on the emotional pictures were more than 50% (see **Table 2**). Specifically, the three groups had an attentional bias to positive and negative pictures characterized by longer total fixation times compared with neutral pictures. Nevertheless, the MDS group paid significantly more attention to negative pictures (*p* = 0.008), and the NDS group had significantly longer fixation duration to positive pictures (*p* = 0.000), compared with neutral pictures.

### Correlation Analysis

Bivariate correlation analyses were conducted to explore whether attentional bias scores were associated with severity of depressive symptoms. **Table 3** shows the zero-order correlation coefficients. Symptoms of depression were positively associated with the bias scores of initial-fixation direction to negative pictures. There were no other statistically significant associations.

**Table 3:** Bivariate correlations between attentional bias scores and depressive symptom.

Attention bias scores	EPDS	
	*R*	*p*
Direction initial gaze—positive	0.153	0.152
Direction initial gaze—negative	0.254*	0.016*
Latency first fixation—positive	−0.103	0.335
Latency first fixation—negative	−0.017	0.872
First fixation duration—positive	−0.047	0.063
First fixation duration—negative	0.178	0.095
Total fixation time—positive	−0.062	0.563
Total fixation time—negative	0.141	0.189

*p < 0.05; EPDS, Edinburgh Postnatal Depression Scale.

## Discussion

The current study is important, not only because it incorporates eye-tracking technology but also because it is the first study to investigate positive and negative image processing biases among women with depression disorder-related symptoms who are in the late stage of pregnancy. The present approach may contribute to the understanding of the possible factors predisposing pregnant women to depression disorders during late pregnancy. These findings may also contribute to the understanding of the nature of depression development and progression and patient responsiveness to treatment in the early stages of depression. Previous studies of cognitive deficits in depressive disorders have tended to focus on clinical populations. This has made it particularly difficult to separate the causal and maintaining factors implicated in the depressive state. Thus, this study looked at the relationship between attentional processing and depressive symptoms within a nonclinical sample.

In the present study, the main finding of relevance to the first hypothesis is that participants with depressive symptoms (MDS and SDS) showed a greater negative attentional bias to negative pictures in the maintenance of gaze, compared with the NDS group. At first gaze, the MDS group tended to focus on positive and negative emotional pictures. However, with prolonged exposure to stimuli, pregnant women with MDS tended to focus more on negative pictures and less on positive pictures. This suggests that the problems disengaging from negative information that are observed in patients with clinical depression might be generalized to those with nonclinical levels of depression-related symptoms. One explanation is that depressed individuals are less able to inhibit negative stimuli during the processing of emotional stimuli and, thus, show a negative attention bias. These results are consistent with previous studies (61, 62).

In addition to paying more attention to negative stimuli, some studies have found an absence of “protective bias” in depressed participants (54, 63). The absence of protective bias has been interpreted as evidence of insensitivity to reward, such that rewarding stimuli fail to capture attention (60). Reduced attention to positive stimuli in pregnant women with MDS might be associated with deficits in positive affect, a factor that appears specific to depressive disorders (64). This tendency to disengage from positive stimuli causes pregnant women with MDS to ignore positive stimuli, making it impossible for them to get out of depression as soon as possible under the guidance of positive substances. This aggravates their negative cognitive bias and makes it difficult for them to recover from the negative mood (65).

Our second hypothesis regarding positive information processing was partially supported by the data. The results suggest that subjects with SDS are able to engage in active coping strategies and may show some traces of resilience in the form of adaptive or compensatory characteristics, such as attentional bias toward positive information. However, these individuals also inevitably show traces of vulnerability. That is, the trace of resilience could be disrupted by MDS in late pregnancy. In addition, the NDS group had an initial attention avoidance tendency toward negative images compared with neutral pictures. This finding is consistent with previous research reporting that healthy participants show an attentional bias toward happy faces (or may avoid sad faces) (66). Interestingly, studies have suggested that attention avoidance occurs when a negative stimulus is presented for a longer period of time (usually greater than 1,000ms) and is in the late stages of cognitive processing (67); however, this is inconsistent with the results of the current study. Attention avoidance is considered to be an anxiety reduction strategy (68). In this study, pregnant women in the NDS group did not show depressive symptoms, but we cannot rule out the presence of state anxiety. Attentional bias is influenced by depression severity and comorbid anxiety (16, 17, 69).

Previous research has suggested that depression-related bias for negative information tends to be more pronounced in the maintenance of attention (gaze duration) than in initial orienting (gaze direction) (70). In the current study, all groups showed a general bias to initially look at positive or negative rather than neutral scenes and had accelerated detection of negative stimuli compared with neutral pictures. These findings suggest that the orienting mechanism is not only selectively biased toward emotional stimuli (indexed by the probability of first fixation) but that it is triggered faster by these stimuli (indexed by shorter saccade latencies) (71). In addition, several studies have suggested that attentional bias related to negative emotions is closely related to attention control and personality traits (72, 73); that is, neuroticism tends to correlate with negative emotional attention bias. In contrast, extraversion tends to correlate with positive emotional attention bias. Specifically, low attentional control might be a risk factor for psychological disorders because those with low attentional control are unable to shift attention away from negative stimuli (switching) and are less able to focus their attention on the task at hand (focusing) (74). Based on results from the dot-probe test, one study argued that a low ability to control attention is a risk factor for attention bias (75). The above theories provide support for the current findings, to some extent. Although the relationship between personality traits and attentional bias is well known, the specific mechanisms underlying attentional bias are not fully elucidated.

The rapid feedback system of the amygdala may also be one of the neural mechanisms underlying early attention enhancement. Our results are initially consistent with neurorelated studies on attentional bias to threat stimuli in clinical anxiety. On the one hand, the amygdala is a brain structure closely related to fear information and fear behavior (76, 77). On the other hand, the relationship between the amygdala and visual cortex is bidirectional in structure and function. This assertion has been supported by the results of magnetic resonance imaging studies. For example, studies have found that the smaller the volume of gray matter in the anterior cingulate gyrus of the amygdala, the smaller the attention bias (78). Another study found that the attentional bias to threatening stimuli is related to the positive functional connection of the amygdala anterior cingulate gyrus; the greater the deviation, the stronger the connection function (79). Therefore, the amygdala anterior cingulate gyrus network, with the amygdala as the core, may be the neural basis for attention-directed acceleration of negative stimuli.

Overall, the results of this study are consistent with our predictions. The severity of depressive symptoms in the third trimester was positively correlated with the initial gaze orientation bias score (i.e., early alertness), but not with the maintenance attention index of negative images. The severity of depressive symptoms was positively associated with total fixation time in clinical depression in a previous study (20). We believe that the initial attention orientation bias score can also be used as an indicator of attention bias in nonclinical depression; this may be one of the differences between clinical depression and nonclinical depression, because one is a state feature and the other is a disease feature. Taken together, these findings suggest the presence of an orientation bias toward negative pictures in late-gestation pregnant women who are considered to be “at risk” of developing perinatal depression. We hypothesize that an evolutionary adaptive shift toward hypervigilant emotion processing in pregnancy could increase the vulnerability of women to depression during late pregnancy. This may explain the increased prevalence of depression in the third trimester compared with early pregnancy (80).

Furthermore, some studies suggest that high risk of perinatal depression is associated with some degree of alexithymia (81, 82) and that alexithymia is a potential risk factor for maternal depression (25). However, other studies suggest that the great emotional experiences associated with pregnancy, childbirth, and motherhood may partially overcome the effects of maternal alexithymia (83). In addition, studies have shown that even the absolute stability of alexithymia may change due to the stress of life events or even pregnancy (84, 85). Still, interest in studying prenatal and postnatal alexithymia is surprisingly low. To the best of our knowledge, there are only two longitudinal studies of expectant mothers, and these have produced inconsistent results (25, 84). Therefore, based on the small number of previous studies and the inconsistent results, we cannot determine the possible impact of alexithymia in the nonclinical sample in this study.

These results are clinically significant and provide further evidence for early attentional correction interventions in women at high risk for perinatal depression. Intervention strategies that load on these resiliency states and traits would likely prove most efficient in preventing future depressive episodes (86). Increasingly, studies are showing that attention bias training to positive information and training in the use of positive coping strategies might be a feasible intervention for some individuals with subclinical depressive tendencies (87–90). If so, in the future, eye-tracking attention tasks may provide objective measures to help understand and identify vulnerable pregnant mothers, allowing prevention of the development of PPD through active attention bias therapy for women with antenatal depression. However, additional work is required to understand the neurocognitive mechanisms underlying the attentional differences seen here; developments in this space will require neuroimaging technology.

Some limitations of the current study are noted. First, the EPDS is a self-report questionnaire, and even though it is commonly used (44), based on the EPDS scores, we are only able to identify women at higher risk of perinatal depression. However, almost 70% of women exceeding the threshold of 10 satisfy the criteria for a diagnosis of depression (12). It is worth noting that EPDS cannot really represent the major depressive disorder group in the clinical perspectives; future studies may consider introducing other scales (e.g., Hamilton Depression Scale) that are widely used in clinical practice to assess the severity of the depression group. Second, sample sizes in eye-movement-tracking studies are usually small. Although the sample size of this study is slightly larger than most eye-movement studies, future research should attempt to replicate these preliminary findings with larger samples. Third, as this study has no comparison group of nonpregnant women, we are limited in the conclusions that can be made about attentional processing of emotional pictures in women generally. Future studies should include nonpregnant women as comparison subjects to determine the associations between sensitivity to emotion-related stimuli and eye-movement indicators in nonpregnant women. Finally, this study was cross-sectional and looked at women in late pregnancy only; thus, it is limited in its ability to confirm a causal relationship between depressive symptom and attentional bias to emotional pictures. A follow-up of this study will investigate these attentional biases after birth.

Taken together, the current findings provide evidence that depressive symptoms during late pregnancy are associated with differential attentional processing of emotion-related information. Women at risk of perinatal depression have a significant bias toward negative stimuli. One interpretation of these findings is that a dysfunctional reward processing system disrupts the development of the maternal response to emotional information during pregnancy. Hypervigilance of emotion processing during pregnancy increases a woman’s susceptibility to depression during late pregnancy. Attention to positive information (or away from negative information) may provide a degree of buffering of emotional responses.

## Data Availability Statement

The datasets generated for this study are available on request to the corresponding author.

## Ethics Statement

This study was carried out in accordance with the recommendations of the research ethics committee of Wenzhou Medical University with written informed consent from all subjects. All subjects gave written informed consent in accordance with the Declaration of Helsinki. The protocol was approved by the research ethics committee of Wenzhou Medical University.

## Author Contributions

JH, KZ, XY, WZ, and MZ designed the study, wrote the protocol, and revised the paper. WT, LC, CB, LX, JZ, WF, QC, and PD conducted literature searches and provided summaries of previous research studies. WT conducted the statistical analysis. CL and YB helped revise the manuscript. WT wrote the first draft of the manuscript, and all authors contributed to and have approved the final manuscript.

## Funding

This work was supported by the Projects of National Science Foundation of China (No. 81873799) and the Natural Science Foundation of Zhejiang Province,China (No. LQ18H090009, LY19H090015).

## Conflict of Interest

The authors declare that the research was conducted in the absence of any commercial or financial relationships that could be construed as a potential conflict of interest.
